# Diversity of spotted fever group rickettsiae and their association with host ticks in Japan

**DOI:** 10.1038/s41598-018-37836-5

**Published:** 2019-02-06

**Authors:** May June Thu, Yongjin Qiu, Keita Matsuno, Masahiro Kajihara, Akina Mori-Kajihara, Ryosuke Omori, Naota Monma, Kazuki Chiba, Junji Seto, Mutsuyo Gokuden, Masako Andoh, Hideo Oosako, Ken Katakura, Ayato Takada, Chihiro Sugimoto, Norikazu Isoda, Ryo Nakao

**Affiliations:** 1 Unit of Risk Analysis and Management, Hokkaido University Research Center for Zoonosis Control, N 20 W 10, Kita-ku, Sapporo, 001-0020 Japan; 2 Laboratory of Parasitology, Faculty of Veterinary Medicine, Graduate School of Infectious Diseases, Hokkaido University, N 18 W 9, Kita-ku, Sapporo, 060-0818 Japan; 3 Hokudai Center for Zoonosis Control in Zambia, School of Veterinary Medicine, University of Zambia, P. O. Box 32379, Lusaka, Zambia; 4 Laboratory of Microbiology, Faculty of Veterinary Medicine, Graduate School of Infectious Diseases, Hokkaido University, N 18 W 9, Kita-ku, Sapporo, 060-0818 Japan; 5 Global Station for Zoonosis Control, Global Institution for Collaborative Research and Education (GI-CoRE), Hokkaido University, N 18 W 9, Kita-ku, Sapporo, 060-0818 Japan; 6 Division of Global Epidemiology, Hokkaido University Research Center for Zoonosis Control, N 20 W 10, Kita-ku, Sapporo, 001-0020 Japan; 7 Division of Bioinformatics, Hokkaido University Research Center for Zoonosis Control, N 20 W 10, Kita-ku, Sapporo, 001-0020 Japan; 8 Precursory Research for Embryonic Science and Technology (PRESTO), Japan Science and Technology Agency, Saitama, 332-0012 Japan; 9 Department of Infection Control, Fukushima Medical University, 1 Hikarigaoka, Fukushima, 960-1295 Japan; 10 Fukushima Institute for Public Health, 16-6 Mitouchi Houkida, Fukushima, 960-8560 Japan; 11 Yamagata Prefectural Institute of Public Health, 1-6-6 Toka-machi, Yamagata, 990-0031 Japan; 12 Kagoshima Prefectural Institute for Environmental Research and Public Health, 11-40 Kinko cho, Kagoshima, 892-0835 Japan; 13 Laboratory of Veterinary Public Health, Joint Faculty of Veterinary Medicine, Kagoshima University, 1-21-24 Korimoto, Kagoshima, 890-0065 Japan; 14 Kumamoto Prefectural Institute of Public-Health and Environmental Science, Uto-shi, Kumamoto, 869-0425 Japan; 15 Division of Collaboration and Education, Hokkaido University Research Center for Zoonosis Control, N 20 W 10, Kita-ku, Sapporo, 001-0020 Japan

## Abstract

Spotted fever group (SFG) rickettsiae are obligate intracellular Gram-negative bacteria mainly associated with ticks. In Japan, several hundred cases of Japanese spotted fever, caused by *Rickettsia japonica*, are reported annually. Other *Rickettsia* species are also known to exist in ixodid ticks; however, their phylogenetic position and pathogenic potential are poorly understood. We conducted a nationwide cross-sectional survey on questing ticks to understand the overall diversity of SFG rickettsiae in Japan. Out of 2,189 individuals (19 tick species in 4 genera), 373 (17.0%) samples were positive for *Rickettsia* spp. as ascertained by real-time PCR amplification of the citrate synthase gene (*gltA*). Conventional PCR and sequencing analyses of *gltA* indicated the presence of 15 different genotypes of SFG rickettsiae. Based on the analysis of five additional genes, we characterised five *Rickettsia* species; *R*. *asiatica*, *R*. *helvetica*, *R*. *monacensis* (formerly reported as *Rickettsia* sp. In56 in Japan), *R*. *tamurae*, and *Candidatus* R. tarasevichiae and several unclassified SFG rickettsiae. We also found a strong association between rickettsial genotypes and their host tick species, while there was little association between rickettsial genotypes and their geographical origins. These observations suggested that most of the SFG rickettsiae have a limited host range and are maintained in certain tick species in the natural environment.

## Introduction

Rickettsiae are obligate intracellular Gram-negative bacteria that belong to the order Rickettsiales in the class Alphaproteobacteria^1^. The members of the genus *Rickettsia* are divided into four main groups: the spotted fever group (SFG), typhus group (TG), transitional group (TRG), and ancestral group (AG)^2^. SFG and AG rickettsiae are mainly associated with ticks, while TG and TRG rickettsiae are associated with other arthropods such as lice, fleas, and mites. TG is composed of *Rickettsia typhi* and *R*. *prowazekii*, while TRG is composed of *R*. *akari*, *R*. *australis*, and *R*. *felis*. Among the tick-borne rickettsiae, AG includes *R*. *bellii* and *R*. *canadensis*. More than 25 species of tick-borne rickettsiae that have been validated so far belong to SFG. Furthermore, the members of SFG rickettsiae have been increasing as many new species have been proposed recently^3–7^.

In Japan, *R*. *japonica* was the first SFG *Rickettsia* discovered in 1984 as the causative agent of Japanese spotted fever (JSF)^8,9^. Since then, several other SFG rickettsiae, namely *R*. *heilongjiangensis*, *R*. *helvetica*, and *R*. *tamurae* have been recognised as etiological agents of human diseases^10–12^. SFG rickettsiae with unknown pathogenicity, such as *R*. *asiatica* and *Candidatus* R. tarasevichiae, have also been reported^13,14^. In addition, several studies conducted in Japan have documented the presence of other *Rickettsia* species/genotypes in animals and questing ticks^15–17^. However, in most cases, only single or a limited number of genes have been analysed, making it difficult to generate an overview of the genetic diversity of SFG rickettsiae, since multiple gene sequencing are recommended in the classification of rickettsial isolates^18^.

The relationship between SFG rickettsiae and their vector tick species has been studied previously. It is evident that some SFG rickettsiae, such as *R*. *rickettsii*, are associated with several different tick species in different genera, while others, such as *R*. *conorii*, are linked to specific tick species^19^. In Japan, *R*. *japonica* is considered to be in the former group since it has been recorded from wide range of tick species including *Dermacentor taiwanensis*, *Haemaphysalis hystricis*, *H*. *cornigera*, *H*. *longicornis*, *H*. *flava*, *H*. *formosensis*, *H*. *megaspinosa*, and *Ixodes ovatus*^20^. On the other hand, vector tick species of other rickettsiae, such as *R*. *asiatica* and *R*. *heilongjiangensis*, which are respectively transmitted by *I*. *ovatus* and *H*. *concinna*, seem to be limited^11,13^.

The aim of the present study was to understand the overall diversity of SFG rickettsiae and their vector tick species in Japan. By collecting questing ticks at more than 100 different sampling sites across Japan, a nationwide cross-sectional study for SFG rickettsiae was conducted. The samples included 19 different tick species covering most of the commonly found species in Japan. Our results indicate that there exist more SFG rickettsiae genotypes than previously known. The information on the relationship between SFG rickettsiae and vector ticks is useful for further characterisation of each rickettsiael member in more detail.

## Results

### Detection of SFG rickettsiae by real-time PCR for *gltA*

Out of 2,189 ticks, 373 (17.0%) samples were positive for *Rickettsia* spp. by citrate synthase gene (*gltA*) real-time PCR (Table 1). Among the 19 different tick species, seven tick species, namely *D*. *taiwanensis*, *H*. *concinna*, *H*. *cornigera*, *H*. *yeni*, *I*. *pavlovskyi*, *I*. *tanuki*, and *I*. *turdus*, were negative for rickettsiae infection. The highest infection rate was observed in *I*. *nipponensis* (80.0%), followed by *H*. *longicornis* (62.8%), *I*. *monospinosus* (58.6%), *H*. *hystricis* (57.8%), *I*. *persulcatus* (34.8%), *A*. *testudinarium* (23.5%), *H*. *megaspinosa* (17.4%), *H*. *flava* (10.2%), *H*. *japonica* (5.1%), *H*. *kitaokai* (4.1%), *H*. *formosensis* (2.8%), and *I*. *ovatus* (1.1%).

**Table 1:** Detection of spotted fever group rickettsiae by real-time and conventional PCR for *gltA* gene.

Tick species	No. tested (Female/Male/Nymph)	Real-time PCR	Conventional PCR
		No. of positive (Female/Male/Nymph) (%)	No. of positive (Female/Male/Nymph) (%)
*A*. *testudinarium*	85 (3/0/82)	20 (1/0/19) (23.5)	16 (1/0/15) (18.8)
*D*. *taiwanensis*	12 (7/5/0)	0	0
*H*. *concinna*	7 (2/5/0)	0	0
*H*. *cornigera*	1 (1/0/0)	0	0
*H*. *flava*	128 (59/65/4)	13 (7/5/1) (10.2)	11 (6/4/1) (8.6)
*H*. *formosensis*	253 (130/122/1)	7 (2/5/0) (2.8)	7 (2/5/0) (2.8)
*H*. *japonica*	78 (50/25/3)	4 (2/2/0) (5.1)	4 (2/2/0) (5.1)
*H*. *hystricis*	64 (42/21/1)	37 (24/13/0) (57.8)	36 (23/13/0) (56.3)
*H*. *kitaokai*	74 (37/36/1)	3 (0/2/1) (4.1)	3 (1/1/1) (4.1)
*H*. *longicornis*	86 (56/26/4)	54 (31/22/1) (62.8)	54 (31/22/1) (62.8)
*H*. *megaspinosa*	201 (106/92/3)	35 (21/14/0) (17.4)	27 (16/11/0) (13.4)
*H*. *yeni*	1 (1/0/0)	0	0
*I*. *monospinosus*	58 (38/20/0)	34 (20/14/0) (58.6)	34 (20/14/0) (58.6)
*I*. *nipponensis*	5 (0/5/0)	4 (0/4/0) (80)	4 (0/4/0) (80)
*I*. *ovatus*	652 (339/313/0)	7 (4/3/0) (1.1)	7 (4/3/0) (1.1)
*I*. *pavlovskyi*	33 (16/17/0)	0	0
*I*. *persulcatus*	446 (220/222/4)	155 (87/68/0) (34.8)	150 (82/68/0) (33.6)
*I*. *tanuki*	2 (1/1/0)	0	0
*I*. *turdus*	3 (3/0/0)	0	0
Total	2,189 (1,111/975/103)	373 (199/152/22) (17.0)	352 (187/147/18) (16.1)

### *gltA* genotyping

**Figure 1:**
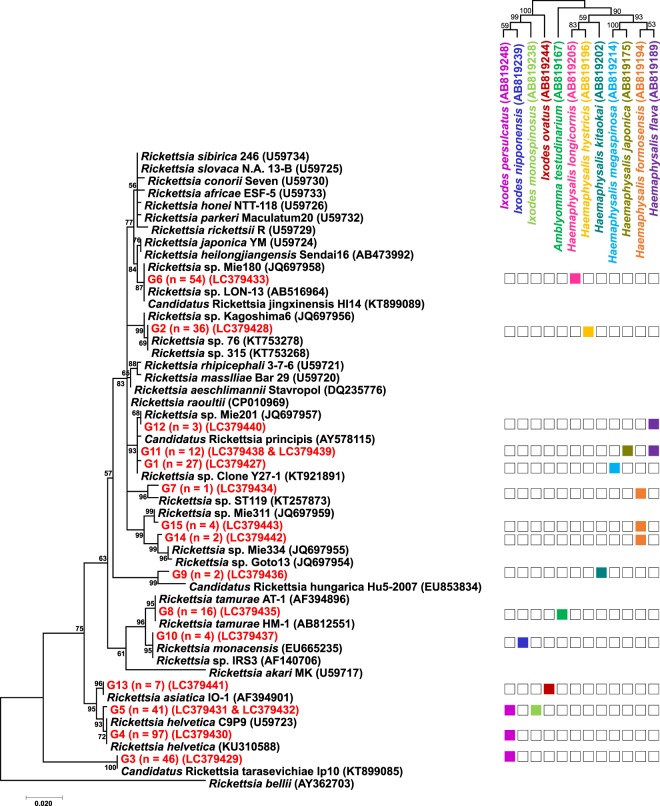
A phylogenetic tree of spotted fever group rickettsiae based on the *gltA* gene sequences (537 bp). The analysis was performed using a maximum likelihood method with the Kimura 2-parameter model. All bootstrap values from 1,000 replications are shown on the interior branch nodes. The sequences detected in this study are indicated in red. The number of samples positive for each genotype is indicated in the parentheses. The simplified tick phylogeny consisting of 12 tick species is indicated on the top right. The colour highlights in the column indicate the presence of infections in each tick species.

Out of 373 samples that tested positive for rickettsiae by real-time PCR for *gltA*, 352 samples yielded amplicons by conventional PCR for *gltA*, while 21 samples did not (Table 1). All the amplicons were successfully sequenced, which resulted in 15 different *gltA* genotypes (Fig. 1 and Table 1). In the present study, the *gltA* genotype is defined as a *gltA* sequence type that is different from the others even by a single nucleotide. The sequence alignment of all 15 *gltA* sequences is provided in Supplementary Fig. S1. All *gltA* genotypes (G1, G2, G6, G7, G9, G11, G12, G14, and G15) detected in the genus *Haemaphysalis* were clustered in the same clade, and five genotypes (G3, G4, G5, G10, and G13) obtained from the genus *Ixodes* were allocated to three different clusters while only one genotype (G8) was linked with the genus *Amblyomma*. (Fig. 1). A total of 13 genotypes were detected in only one single tick species, while two genotypes (G5 and G11) were detected in two different tick species: G5 was recovered from *I*. *persulcatus* and *I*. *monospinosus*, and G11 was from *H*. *japonica* and *H*. *flava*. Three tick species harboured multiple *gltA* genotypes: *I*. *persulcatus*, *H*. *formosensis*, and *H*. *flava* had 3, 3, and 2 different *gltA* genotypes, respectively.

### Geographic information on *gltA* genotypes and host ticks

Table 2 represents the relationship between *gltA* genotypes and their geographical origins. Out of 15 genotypes, 11 genotypes (G1, G2, G3, G4, G5, G6, G8, G10, G11, G12, and G15) were detected from multiple geographical regions. The other 4 genotypes (G7, G9, G13, and G14) were detected in only one single tick species from a single region. G7 and G14 were detected in *H*. *formosensis* from Kyusyu region, while G9 and G13 were in *H*. *kitaokai* from Kansai region and *I*. *ovatus* from Tohoku region, respectively (Fig. 1). The present study employed *H*. *formosensis* and *H*. *kitaokai* from three different regions and *I*. *ovatus* collected from five different regions (see Supplementary Table S1).

**Table 2:** Host ticks and geographic origin of 15 *gltA* genotypes of spotted fever group rickettsiae.

*gltA* genotype	Tick species	No. of positive/No. tested (%)						
		Hokkaido	Tohoku	Chubu	Kansai	Kyushu	Okinawa	Total
G1	*H*. *megaspinosa*	5/94 (5.3)	0/2 (0)	−	14/97 (14.4)	8/8 (100)	-	27/201 (13.4)
G2	*H*. *hystricis*	−	−	−	5/8 (62.5)	31/53 (58.5)	0/3 (0)	36/64 (56.3)
G3	*I*. *persulcatus*	44/376 (11.7)	2/51 (3.9)	0/11 (0)	0/8 (0)	−	−	46/446 (10.3)
G4	*I*. *persulcatus*	96/376 (25.5)	0/51 (0)	1/11 (9.1)	0/8 (0)	−	−	97/446 (21.7)
G5	*I*. *persulcatus*	7/376 (18.6)	0/51 (0)	0/11 (0)	0/8 (0)	−	−	7/446 (1.6)
G5	*I*. *monospinosus*	−	34/58 (58.6)	−	−	−	−	34/58 (58.6)
G6	*H*. *longicornis*	0/4 (0)	0/2 (0)	5/5 (100)	49/61 (80.3)	0/14 (0)	−	54/86 (62.8)
G7	*H*. *formosensis*	−	−	−	0/34 (0)	1/216 (0.5)	0/3 (0)	1/253 (0.4)
G8	*A*. *testudinarium*	−	−	−	11/64 (17.2)	4/20 (20.0)	1/1 (100)	16/85 (18.8)
G9	*H*. *kitaokai*	−	−	−	2/43 (4.7)	0/14 (0)	0/17 (0)	2/74 (2.7)
G10	*I*. *nipponensis*	—	2/3 (66.7)	−	2/2 (100)	−	−	4/5 (80.0)
G11	*H*. *japonica*	2/49 (4.1)	2/27 (7.4)	−	0/2 (0)	−	−	4/78 (5.1)
G11	*H*. *flava*	−	3/28 (10.7)	−	4/71 (5.6)	1/29 (3.4)	−	8/128 (6.3)
G12	*H*. *flava*	−	1/28 (3.6)	−	2/71 (2.8)	0/29 (0)	−	3/128 (2.3)
G13	*I*. *ovatus*	0/463 (0)	7/163 (4.3)	0/10 (0)	0/15 (0)	0/1 (0)	−	7/652 (1.1)
G14	*H*. *formosensis*	−	−	−	0/34 (0)	2/216 (0.9)	0/3 (0)	2/253 (0.8)
G15	*H*. *formosensis*	−	−	−	1/34 (2.9)	3/216 (1.4)	0/3 (0)	4/253 (1.6)

−, This tick species was not collected in the region.

### Multiple genes sequencing

To further characterise *Rickettsia* spp. based on five other genes; outer membrane protein A gene (*ompA*), outer membrane protein B gene (*ompB*), 17-kDa common antigen gene (*htrA*), surface cell antigen-4 (*sca4*), and 16S ribosomal RNA gene (16S rRNA), PCR analyses were conducted on the selected samples of each *gltA* genotype. A total of 57 samples were employed in this analysis. We selected more than two samples from each genotype except for G7 which was found in only one sample (Table 3). The samples with higher rickettsial burden were selected based on the results of *gltA* real-time PCR. The mean rickettsial burden in the template DNA ranged from 2.3E + 2 to 2.1E + 4 copies/μL (Table 3). The *htrA* gene was successfully amplified and sequenced for all *gltA* genotypes. Although 16S rRNA PCR gave amplicons in all *gltA* genotypes, the following sequencing analysis revealed that rickettsial 16S rRNA gene sequences were obtained in only 12 *gltA* genotypes. The *ompB*, *ompA* and *sca4* genes were amplified and sequenced in 11, five and six different *gltA* genotypes, respectively. All genes were successfully sequenced in two *gltA* genotypes (G6 and G7). Four genes were successfully amplified in six *gltA* genotypes (G1, G2, G5, G8, G10, and G11), and three genes were amplified in four *gltA* genotypes (G3, G4, G9, and G13). Only the *htrA* gene was amplified in three *gltA* genotypes (G12, G14, and G15) (Table 3). The sequencing analysis of the amplified products revealed that there were no sequence differences in any of the genes in the samples with the same *gltA* genotypes. The sequence types obtained from each *gltA* genotype were different from each other. The sequence identity with the closest *Rickettsia* species are provided in Supplementary Table S2.

**Table 3:** Results of PCR amplification for the *ompA*, *ompB*, *htrA*, *sca4* and 16S rRNA genes.

*gltA* genotype	Tick species	No. tested	Mean rickettsial burden (copies/µl)*	PCR amplification				
				*ompA*	*ompB*	*htrA*	*sac4*	16S rRNA
G1	*H*. *megaspinosa*	2	7.9E + 3	−	+	+	+	+
G2	*H*. *hystricis*	2	1.1E + 4	−	+	+	+	+
G3	*I*. *persulcatus*	4	8.7E + 3	+	−	+	−	+
G4	*I*. *persulcatus*	3	8.4E + 3	−	+	+	−	+
G5	*I*. *persulcatus*	6	1.3E + 3	−	−	+	−	−
G5	*I*. *monospinosus*	6	2.3E + 2	−	+	+	+	+
G6	*H*. *longicornis*	2	2.4E + 3	+	+	+	+	+
G7	*H*. *formosensis*	1	1.0E + 4	+	+	+	+	+
G8	*A*. *testudinarium*	3	2.1E + 4	+	+	+	−	+
G9	*H*. *kitaokai*	2	1.6E + 4	−	+	+	−	+
G10	*I*. *nipponensis*	2	3.0E + 3	+	+	+	−	+
G11	*H*. *japonica*	3	2.6E + 3	−	+	+	+	+
G11	*H*. *flava*	7	1.8E + 3	−	−	+	−	−
G12	*H*. *flava*	3	1.2E + 3	−	−	+	−	−
G13	*I*. *ovatus*	5	2.5E + 3	−	+	+	−	+
G14	*H*. *formosensis*	3	3.6E + 3	−	−	+	−	−
G15	*H*. *formosensis*	3	1.3E + 3	−	−	+	−	−

+, Amplified; −, Not amplified. *The mean copy number of rickettsial *gltA* gene in the template DNA was calculated by *gltA* real-time PCR.

### Species classification of SFG rickettsiae

**Figure 2:**
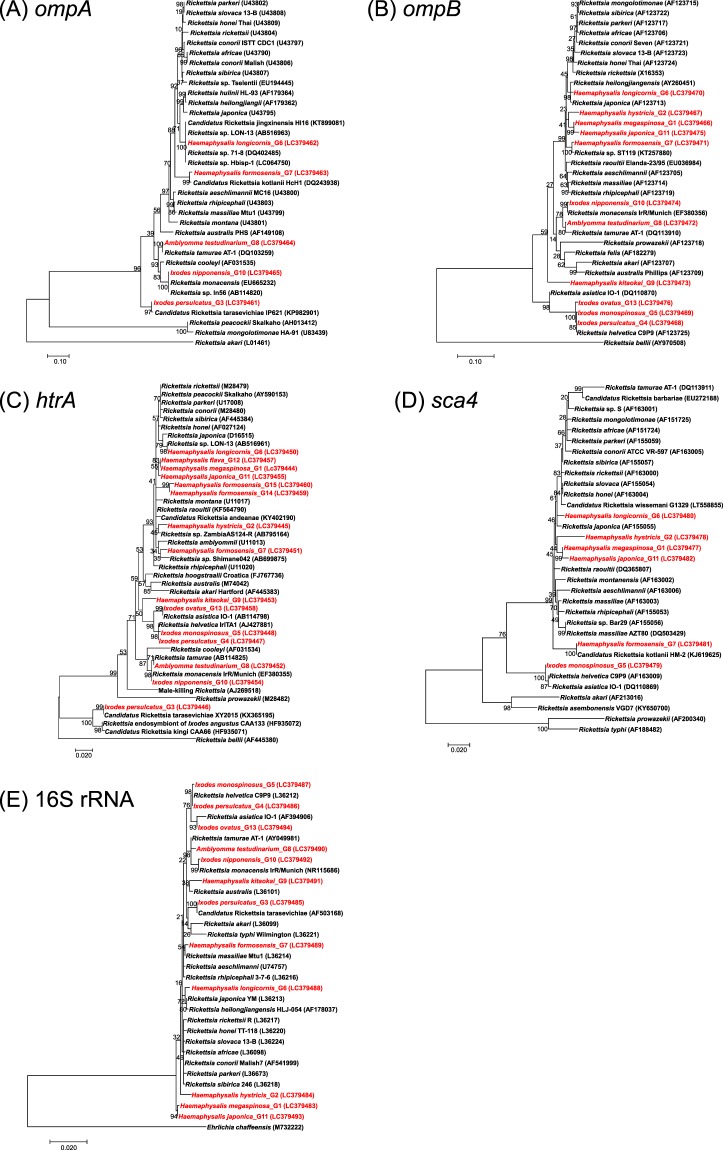
Phylogenetic trees based on the sequences of the *ompA* (493 bp) (**A**), *ompB* (780 bp) (**B**), *htrA* (465 bp) (**C**), *sca4* (887 bp) (**D**), and 16S rRNA (1,245 bp) (**E**) genes. The analyses were performed using a maximum likelihood method with the Kimura 2-parameter model. All bootstrap values from 1,000 replications are shown on the interior branch nodes. The sequences obtained in this study are shown in red.

Phylogenetic trees inferred from *ompA*, *ompB*, *sca4*, *htrA*, and 16S rRNA analysis are shown in Fig. 2. G4 and G5 formed a distinct cluster with *R*. *helvetica* in all trees when sequences were available and thus were identified as *R*. *helvetica*. Being supported by more than three trees, G13, G10, G8, and G3 were identified as *R*. *asiatica*, *R*. *monacensis* (former *Rickettsia* sp. In56), and *R*. *tamurae*, and *Candidatus* R. tarasevichiae, respectively (Fig. 2). The other nine *gltA* genotypes could not be classified into specific species due to a lack of consensus between the trees and/or absence of sequences from previously validated rickettsial species in the same phylogenetic clusters.

## Discussion

The present study included a total of 2,189 individual ticks collected at 114 different sampling sites in six regions of Japan for the screening of SFG rickettsiae. Our nationwide sampling enabled us to collect as many as 19 different tick species from four genera, most of which were common tick species prevalent in Japan. A first screening test using *gltA* real-time PCR revealed that 17.0% (373 out of 2,189) of the ticks were infected with SFG rickettsiae. This infection rate was comparative to the results of an earlier study where 21.9% (181 out of 827) of the ticks, including 10 different species collected from central (Shizuoka, Mie, and Wakayama prefectures) and southern (Kagoshima, Nagasaki, and Okinawa prefectures) parts of Japan, were positive for SFG rickettsiae^16^. Another nationwide survey conducted in 5 prefectures (Chiba, Hokkaido, Kochi, Tokushima, and Toyama prefectures) including JSF endemic areas reported an overall positive rate for SFG rickettsiae to be 25.8% (186 out of 722) in 10 different tick species^21^.

We determined partial sequences of the *gltA* gene of SFG rickettsiae by conventional PCR, which was previously designed to characterise SFG rickettsiae in Japan^16^. Based on the sequences of the *gltA* gene obtained from 352 ticks, the SFG rickettsiae detected in the present study were provisionally divided into 15 genotypes. In the molecular classification of SFG rickettsiae, the analysis of multiple genes commonly used by other researchers is a prerequisite^18^. Therefore, we attempted to obtain the sequences of five additional genes, *ompA*, *ompB*, *htrA*, *sca4*, and 16S rRNA. These efforts lead to the identification of four validated rickettsial species, namely *R*. *asiatica*, *R*. *helvetica*, *R*. *monacensis*, and *R*. *tamurae*, and the provisional species *Candidatus* R. tarasevichiae (Fig. 2).

Prior to this study, there was no official report of the presence of *R*. *monacensis* in Japan. A recent study indicated that *Rickettsia* sp. In56, a rickettsial strain reported from ticks in Japan^21^, might be a synonymous of *R*. *monacensis*^22^. Although several isolates of *Rickettsia* sp. In56 have been obtained from Japanese ticks^23^, lack of their sequence information prevents a direct comparison between *Rickettsia* sp. In56 and *R*. *monacensis* reported elsewhere. Nevertheless, the sequence analysis of multiple genes (*gltA*, *ompA*, *ompB*, *htrA*, and 16S rRNA) conducted in the present study confirmed the presence of *R*. *monacensis* in Japan (Figs 1 and 2). *R*. *monacensis* was initially isolated from *I*. *ricinus* collected from the English Garden in Germany using ISE6 cells^24^ and has been detected from the same tick species in Europe and neighbouring countries^25–29^. *I*. *nipponensis* and *I*. *sinensis* are considered as main vectors of *R*. *monacensis* in China and Korea, respectively^30,31^. In our study, *R*. *monacensis* was detected from four *I*. *nipponensis* samples collected in the Tohoku and Kansai regions, while none of the other tick species carried *R*. *monacensis* (Fig. 1 and Table 2). These results may suggest the relatively wide distribution of *R*. *monacensis* and a strong association of *R*. *monacensis* with *I*. *nipponensis* in Japan. This SFG rickettsiae caused Mediterranean spotted fever-like symptoms in humans in several countries^32,33^. More recently, the agent was isolated from the blood of a patient with an acute febrile illness in Korea^22^. Thus, clinicians should be aware of *R*. *monacensis* as a possible cause of non-JSF rickettsiosis in Japan.

Although we tried to characterise SFG rickettsiae with each prospective *gltA* genotype in further detail by sequencing five additional rickettsial genes, *ompA*, *ompB*, *htrA*, *sca4*, and 16S rRNA, the amplification was not successful for some genes (Table 3). The *ompA* and *sca4* genes were amplified only from one third of the tested *gltA* genotypes. Considering the relatively high rickettsial abundance in the tested samples (Table 3), PCR failure is either because some of the SFG rickettsiae lack these genes as shown in TG rickettsiae that do not possess *ompA* gene^34^, or because there are nucleotide mismatches in the primer annealing sites. PCR failures of variable genes such as *ompA*, *ompB*, and *sca4* are common issues in the genetic characterisation of SFG rickettsiae^34,35^. Thus further attempts including the development of universal primers and/or bacterial isolation followed by whole genome sequencing are required to determine the phylogenetic positions of uncharacterised *Rickettsia* spp.

In a previous nationwide survey of SFG rickettsiae conducted in Japan, Gaowa *et al*.^16^ classified the detected rickettsiae (n = 181) into five groups (groups 1–5) based on the *gltA* sequences^16^. Groups 1 and 2 were respectively identified as *R*. *japonica* and *R*. *tamurae*, whereas groups 3, 4, and 5, showing high sequence similarity with *Rickettsia* sp. LON-13, *R*. *raoultii*, and *Candidatus* R. principis, respectively, were not classified as validated rickettsial species^16^. In agreement with their report, we detected *gltA* sequences corresponding to groups 3 (G6), 4 (G2), and 5 (G1, G11, G12, G14, and G15) (Fig. 2). Unfortunately, limited information is available about these uncharacterised *Rickettsia* spp. In our study, G6 and G2 were respectively detected in *H*. *longicornis* and *H*. *hystricis* with high infection rates (62.8% and 57.8%, respectively) (Table 1), warranting further studies on the effect of these infections for the survival and reproductive fitness of their hosts.

We detected two *gltA* genotypes (G7 and G9), which were allocated into distinct clusters from *Rickettsia* spp. previously reported from Japan (Fig. 2). G7 and G9 showed the highest *gltA* sequence similarity with *Rickettsia* spp. reported from Kenya (KT257873) and Hungary (EU853834), respectively. *Rickettsia* sp. reported from Kenya was detected in *Rhipicephalus maculatus*^36^, while the one from Hungary was detected in *H*. *inermis* and provisionally named as *Candidatus* R. hungarica^37^. Since the sequences of other genes were not available from those *Rickettsia* spp., it was difficult to evaluate the degree of genetic relatedness in more detail. Nonetheless, the presence of closely related species in two geographically remote areas may indicate the worldwide distribution of these poorly characterised SFG rickettsiae. Since the present study provided the sequences of multiple genes of those rickettsiae, the information is useful in the classification of SFG rickettsiae.

In the present study, we found a strong association between rickettsial genotypes and their host tick species; 13 out of 15 *gltA* genotypes were detected in only one single tick species (Fig. 1 and Table 3). Furthermore, there was minimal geographical restriction for the 11 *gltA* genotypes that were recovered from multiple geographical regions (Table 2). These observations may indicate that most of the SFG rickettsiae species are found in ticks but not in vertebrate hosts in the natural environment. However, further examinations are needed to confirm this hypothesis by observing transstadial and transovarial transmission of these SFG rickettsiae in ticks. The effect of these rickettsial infections on tick physiology and reproduction remains to be elucidated.

Although the sampling was conducted at several JSF-endemic areas in Mie, Kagoshima, and Kumamoto prefectures, none of the ticks were infected with *R*. *japonica*. Considering the low level of genomic plasticity within *R*. *japonica* isolates^38^, it was hardly expected that a real-time PCR assay for *gltA* would result in false-negatives. The positive rate of *R*. *japonica* infection in the questing ticks was as low as 0.86% (18 out of 2,099), even in endemic areas as is the case in Shimane prefecture^39^. Collectively, the failure in detection of *R*. *japonica* might be partly attributed to the sample selection procedure with which only a maximum of 10 individual ticks per species, stage/sex, and site were tested for SFG rickettsiae infection. Therefore, it should be noted that the present study might not fully disclose the diversity of SFG rickettsiae in Japan, which warrants further investigations by employing a larger number of samples.

The present study has extended our knowledge on the diversity of SFG rickettsiae prevalent in Japan. In addition to previously recognised rickettsial species such as *R*. *asiatica*, *R*. *helvetica*, *R*. *monacensis* (formerly reported as *Rickettsia* sp. In56), *R*. *tamurae*, and *Candidatus* R. tarasevichiae, several uncharacterised *Rickettsia* spp. including ones showing high similarities with those designated as novel *Rickettsia* spp. detected in geographically remote countries such as Kenya and Hungary were discovered. A strong association between rickettsial genotypes and their host ticks suggests a long-term relationship between SFG rickettsiae and ticks. Further investigations on the potential roles of these SFG rickettsiae on ticks are required to understand the mechanisms underlying widespread existence of genetically variable rickettsiae in ticks.

## Materials and Methods

### Sample collection

**Figure 3:**
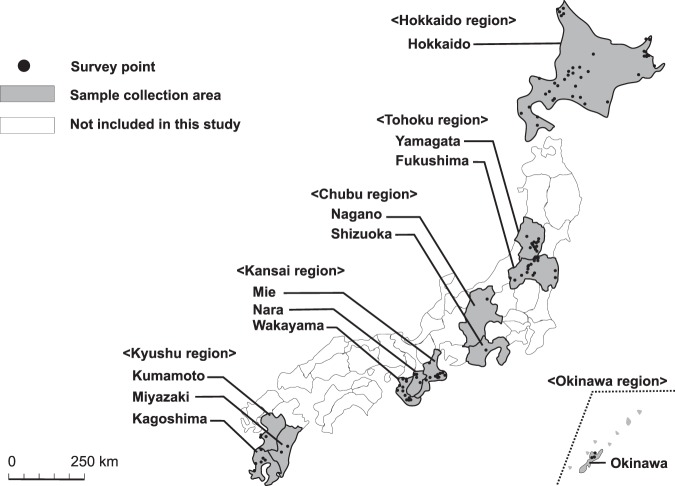
Sample collection sites.

Ticks were collected by flagging a flannel cloth over the vegetation during the period of tick activity (between April 2013 and March 2016) at 114 different sampling sites in 12 different prefectures. The sampling sites were categorised into geographical blocks: Hokkaido (Hokkaido prefecture), Tohoku (Yamagata and Fukushima prefectures), Chubu (Nagano and Shizuoka prefectures), Kansai (Mie, Nara, and Wakayama prefectures), Kyushu (Kumamoto, Miyazaki, and Kagoshima prefectures), and Okinawa (Okinawa prefecture) (Fig. 3). All field-collected ticks were transferred to small Petri dishes and preserved in an incubator at 16 °C until use.

### Tick species identification

Tick species were identified morphologically using standard keys under a stereomicroscope^40,41^. When more than 10 ticks with the same species and stage/sex were collected from the same sampling sites, a maximum of 10 individual ticks were analysed per species, stage/sex and site. A total of 2,189 individuals (103 nymphs and 2,086 adults) in four genera were examined in this study. These included one species in the genus *Amblyomma* (*A*. *testudinarium*, n = 85), one species in the genus *Dermacentor* (*D*. *taiwanensis*, n = 12), 10 species in the genus *Haemaphysalis* (*H*. *concinna*, n = 7; *H*. *cornigera*, n = 1; *H*. *flava*, n = 128; *H*. *formosensis*, n = 253; *H*. *japonica*, n = 78; *H*. *hystricis*, n = 64; *H*. *kitaokai*, n = 74; *H*. *longicornis*, n = 86; and *H*. *megaspinosa*, n = 201; *H*. *yeni*, n = 1) and 7 species in the genus *Ixodes* (*I*. *monospinosus*, n = 58; *I*. *nipponensis*, n = 5; *I*. *ovatus*, n = 652; *I*. *pavlovskyi*, n = 33; *I*. *persulcatus*, n = 446; *I*. *tanuki*, n = 2; and *I*. *turdus*, n = 3). Out of 2,189 ticks, 975, 1,111, and 103 were male, female, and nymph, respectively.

### DNA extraction

Ticks were individually washed with 70% ethanol followed by washing with sterile PBS twice, then homogenised in 100 μL of high glucose Dulbecco’s Modified Eagle Medium (DMEM)  (Gibco, Life Technologies) by using Micro Smash MS-100R (TOMY, Tokyo, Japan) for 30 sec at 3,000 rpm as described previously^42^. DNA was extracted from 50 μL of the tick homogenate using a blackPREP Tick DNA/RNA Kit (Analytikjena, Germany) according to the manufacturer’s instructions, while the other half was kept at −80 °C for future bacterial isolation.

### Real-time PCR

All samples were first screened for rickettsial *gltA* using real-time PCR to detect SFG and TG rickettsiae as described previously^43^. The primers and probes used are shown in Table 4. Reactions were performed in a 20.0 μL of reaction mixture containing 10.0 μL of THUNDERBIRD Probe qPCR Mix (Toyobo, Osaka, Japan), 300 nM of each primer, 200 nM of probe, 5.0 μL of template DNA, and distilled water. The reaction was carried out in a C1000 Thermal Cycler with a CFX96 Real-Time PCR Detection System (BioRad Laboratories, Hercules, CA) at conditions of 50 °C for 3 min, 95 °C for 1 min, and 40 cycles of 95 °C for 15 sec and 60 °C for 1 min. Each run included a blank control and serially diluted plasmid standards (10^6^, 10^4^, and 10^2^ copies/reaction) as described previously^35^.

**Table 4:** Primers uesd in the present study.

Primer	Sequence (5′-3′)	Target gene	Annealing temperature (°C)	Amplicon size (bp)	Reference
CS-F	TCGCAAATGTTCACGGTACTTT	citrate synthase gene (*gltA*)	60	74	Steno *et al*.^43^
CS-R	TCGTGCATTTCTTTCCATTGTG				
CS-P	TGCAATAGCAAGAACCGTAGGCTGGATG				
gltA_Fc	CGAACTTACCGCTATTAGAATG	citrate synthase gene (*gltA*)	55	580	Gaowa *et al*.^16^
gltA_Rc	CTTTAAGAGCGATAGCTTCAAG				
Rr.190.70p	ATGGCGAATATTTCTCCAAAA	outer membrane A gene (*ompA*)	48	542	Regnery *et al*.^44^
Rr.190.602n	AGTGCAGCATTCGCTCCCCCT				
120_2788	AAACAATAATCAAGGTACTGT	outer membrane B gene (*ompB*)	48	816	Roux and Raoult^45^
120_3599	TACTTCCGGTTACAGCAAAGT				
17k_5	GCTTTACAAAATTCTAAAAACCATATA	17-kDa common antigen gene (*htrA*)	52	550	Labruna *et al*.^47^
17k_3	TGTCTATCAATTCACAACTTGCC				
Rick_16S_F3	ATCAGTACGGAATAACTTTTA	16S ribosomal RNA gene (16S rRNA)	52	1328	Anstead *et al*.^48^
Rick_16S_F4	TGCCTCTTGCGTTAGCTCAC				
rrs2_seq_1	AGGCCTTCATCACTCACTCG*				This study
rrs2_seq_2	CTACACGCGTGCTACAATGG*				
D1f	ATGAGTAAAGACGGTAACCT	surface cell antigen-4 (*sca4*)	50	928	Sekeyova *et al*.^46^
D928r	AAGCTATTGCGTCATCTCCG				
sca4_seq_1	GCCGGCTATTTCTATTGATTC*				This study
sca4_seq_2	TGCAAGCGATCTTAGAGCAA*				This study

*The primers were used for sequencing.

### Conventional PCR

All the samples that were positive for *gltA* by real-time PCR were further characterised by conventional PCR targeting an approximately 580 bp sequence of the *gltA* gene using the primers gltA_Fc and gltA_Rc (Table 4)^16^. The PCR was carried out in a 25.0 μL reaction mixture containing 12.5 μL of 2 × KAPA blood PCR Kit (KAPA Biosystems, USA), 200 nM of each primer, 2.0 μL of DNA template, and sterile water. The reactions were performed at 95 °C for 5 min; followed by 45 cycles of 95 °C for 30 sec, 55 °C for 30 sec, and 72 °C for 40 sec; and 72 °C for 5 min. PCR products were electrophoresed at 100 V in a 1.2% agarose gel for 25 min. DNA from the *R*. *japonica* YH strain and sterile water were included in each PCR run as positive and negative controls, respectively. For the selected samples from each *gltA* genotype (n = 57), additional PCR assays were conducted based on five genes: *ompA*, *ompB*, *sca4*, *htrA*, and 16S rRNA. The primer sets used for each assay are shown in Table 4^44–48^. PCR conditions were the same as mentioned above except for the annealing temperature (48 °C for *ompA* and *ompB* PCRs, 52 °C for 16S rRNA and *htrA* PCRs, and 50 °C for *sca4* PCR).

### Sequencing

The amplified PCR products were purified using a Wizard® SV Gel and PCR Clean-Up System Kit (Promega, USA). Sanger sequencing was conducted using the BigDye Terminator version 3.1 Cycle Sequencing Kit (Applied Biosystems, Foster City, CA) and an ABI Prism3130x genetic analyser according to the manufacturers’ instructions. The sequences data were assembled using ATGC software version 6.0.4 (GENETYX, Tokyo, Japan). The sequences obtained were submitted to the DNA Data Bank of Japan (DDBJ) (http://www.ddbj.nig.ac.jp) under accession numbers (*gltA*: LC379427-LC379443; *ompA*: LC379461-LC379465; *ompB*: LC379466-LC379476; *htrA*: LC379444-LC379460; *sca4*: LC379477-LC379482; 16S rRNA: LC379483-LC379494).

### Phylogenetic analysis

The nucleotide sequences obtained were aligned with representative sequences of known rickettsial species available on GenBank using ClustalW 1.6 as implemented in MEGA 7^49^. After manual modification of the alignments, phylogenetic trees were constructed using the maximum likelihood method using Kimura 2-parameter with bootstrap tests of 1,000 replicates via MEGA. *R*. *bellii* was included as an outgroup for the bases of the trees for *gltA*, *ompB*, and *htrA*, while *R*. *typhi*, *R*. *akari*, and *Ehrlichia chaffeensis* were used as outgroups for *sca4*, *ompA*, 16S rRNA, respectively. In order to generate a phylogenetic tree of tick species that was positive for *Rickettsia* spp., partial nucleotide sequences of mitochondrial 16S rRNA gene obtained from GenBank were used.

## Supplementary information

SUPPLEMENTARY INFORMATION

## Data Availability

All data discussed in the manuscript is included in the paper.
