# Supplementary material for: Diversity of spotted fever group rickettsiae and their association with host ticks in Japan
Source: Sci Rep. 2019 Feb 6;9:1500. doi: 10.1038/s41598-018-37836-5 (PMC6365641; doi:10.1038/s41598-018-37836-5)
Supplement: Supplementary file 1 — SUPPLEMENTARY INFORMATION [file 41598_2018_37836_MOESM1_ESM.docx]

**SUPPLEMENTARY INFORMATION**

**Diversity of spotted fever group rickettsiae and their association with host ticks in Japan**

May June Thu^1,2^, Yongjin Qiu^3^, Keita Matsuno^4,5^, Masahiro Kajihara^6^, Akina Mori-Kajihara^6^, Ryosuke Omori^7,8^, Naota Monma^9^, Kazuki Chiba^10^, Junji Seto^11^, Mutsuyo Gokuden^12^, Masako Andoh^13^, Hideo Oosako^14^, Ken Katakura^2^, Ayato Takada^5,6^, Chihiro Sugimoto^5,15^, Norikazu Isoda^1,5^, Ryo Nakao^2,*^

^1^Unit of Risk Analysis and Management, Hokkaido University Research Center for Zoonosis Control, N 20 W 10, Kita-ku, Sapporo 001-0020, Japan

^2^Laboratory of Parasitology, Graduate School of Veterinary Medicine, Hokkaido University, N 18 W 9, Kita-ku, Sapporo 060-0818, Japan

^3^Hokudai Center for Zoonosis Control in Zambia, School of Veterinary Medicine, University of Zambia, P. O. Box 32379, Lusaka, Zambia

^4^Laboratory of Microbiology, Graduate School of Veterinary Medicine, Hokkaido University, N 18 W 9, Kita-ku, Sapporo 060-0818, Japan

^5^Global Station for Zoonosis Control, Global Institution for Collaborative Research and Education (GI-CoRE), Hokkaido University, N 18 W 9, Kita-ku, Sapporo 060-0818, Japan

^6^Division of Global Epidemiology, Hokkaido University Research Center for Zoonosis Control, N 20 W 10, Kita-ku, Sapporo 001-0020, Japan

^7^Division of Bioinformatics, Hokkaido University Research Center for Zoonosis Control, N 20 W 10, Kita-ku, Sapporo 001-0020, Japan

^8^Precursory Research for Embryonic Science and Technology (PRESTO), Japan Science and Technology Agency, Saitama 332-0012, Japan

^9^Department of Infection Control, Fukushima Medical University, 1 Hikarigaoka, Fukushima

960-1295, Japan

^10^Fukushima Institute for Public Health, 16-6 Mitouchi Houkida, Fukushima 960-8560, Japan

^11^Yamagata Prefectural Institute of Public Health, 1-6-6 Toka-machi, Yamagata

990-0031, Japan

^12^Kagoshima Prefectural Institute for Environmental Research and Public Health, 11-40 Kinko cho, Kagoshima 892-0835, Japan

^13^Laboratory of Veterinary Public Health, Joint Faculty of Veterinary Medicine, Kagoshima University, 1-21-24 Korimoto, Kagoshima 890-0065, Japan

^14^Kumamoto Prefectural Institute of Public-Health and Environmental Science, Uto-shi, Kumamoto 869-0425, Japan

^15^Division of Collaboration and Education, Hokkaido University Research Center for Zoonosis Control, N 20 W 10, Kita-ku, Sapporo 001-0020, Japan

***Corresponding author**: Ryo Nakao, ryo.nakao@vetmed.hokudai.ac.jp

**Supplementary Table S1: Geographic information on *Rickettsia* *gltA* genotypes and tick species.**

| Region | Prefecture | *Rickettsia* *gltA* genotype | | | | | | | | | | | | | | | Tick species | | | | | | | | | | | | | | | | | | |
| --- | --- | --- | --- | --- | --- | --- | --- | --- | --- | --- | --- | --- | --- | --- | --- | --- | --- | --- | --- | --- | --- | --- | --- | --- | --- | --- | --- | --- | --- | --- | --- | --- | --- | --- | --- |
|  |  | G1 | G2 | G3 | G4 | G5 | G6 | G7 | G8 | G9 | G10 | G11 | G12 | G13 | G14 | G15 | Ate | Dta | Hcn | Hcg | Hfl | Hfo | Hja | Hhy | Hki | Hlo | Hme | Hye | Imo | Ini | Iov | Ipe | Ipa | Ita | Itu |
| Hokkaido | Hokkaido | 5 | 0 | 44 | 96 | 7 | 0 | 0 | 0 | 0 | 0 | 2 | 0 | 0 | 0 | 0 | 0 | 0 | 7 | 0 | 0 | 0 | 49 | 0 | 0 | 4 | 94 | 0 | 0 | 0 | 463 | 376 | 33 | 1 | 0 |
| Tohoku | Yamagata | 0 | 0 | 0 | 0 | 23 | 0 | 0 | 0 | 0 | 1 | 0 | 1 | 6 | 0 | 0 | 0 | 0 | 0 | 0 | 6 | 0 | 3 | 0 | 0 | 0 | 1 | 0 | 35 | 1 | 91 | 8 | 0 | 1 | 0 |
|  | Fukushima | 0 | 0 | 2 | 0 | 11 | 0 | 0 | 0 | 0 | 1 | 5 | 0 | 1 | 0 | 0 | 0 | 3 | 0 | 0 | 24 | 0 | 22 | 0 | 17 | 2 | 1 | 0 | 23 | 2 | 72 | 43 | 0 | 0 | 0 |
| Chubu | Nagano | 0 | 0 | 0 | 1 | 0 | 0 | 0 | 0 | 0 | 0 | 0 | 0 | 0 | 0 | 0 | 0 | 0 | 0 | 0 | 0 | 0 | 0 | 0 | 0 | 0 | 0 | 0 | 0 | 0 | 10 | 11 | 0 | 0 | 0 |
|  | Shizuoka | 0 | 0 | 0 | 0 | 0 | 5 | 0 | 0 | 0 | 0 | 0 | 0 | 0 | 0 | 0 | 0 | 0 | 0 | 0 | 0 | 0 | 0 | 0 | 0 | 5 | 0 | 0 | 0 | 0 | 0 | 0 | 0 | 0 | 0 |
| Kansai | Mie | 10 | 0 | 0 | 0 | 0 | 22 | 0 | 10 | 2 | 2 | 4 | 2 | 0 | 0 | 1 | 61 | 0 | 0 | 0 | 64 | 28 | 0 | 1 | 35 | 26 | 69 | 0 | 0 | 2 | 4 | 0 | 0 | 0 | 2 |
|  | Nara | 3 | 0 | 0 | 0 | 0 | 0 | 0 | 0 | 0 | 0 | 0 | 0 | 0 | 0 | 0 | 1 | 1 | 0 | 0 | 1 | 0 | 0 | 0 | 8 | 0 | 7 | 0 | 0 | 0 | 9 | 8 | 0 | 0 | 0 |
|  | Wakayama | 1 | 5 | 0 | 0 | 0 | 27 | 0 | 1 | 0 | 0 | 0 | 0 | 0 | 0 | 0 | 2 | 7 | 0 | 1 | 6 | 6 | 2 | 7 | 0 | 34 | 2 | 1 | 0 | 0 | 2 | 0 | 0 | 0 | 0 |
| Kyushu | Kumamoto | 0 | 3 | 0 | 0 | 0 | 0 | 0 | 1 | 0 | 0 | 0 | 0 | 0 | 1 | 2 | 1 | 0 | 0 | 0 | 0 | 148 | 0 | 13 | 0 | 0 | 0 | 0 | 0 | 0 | 0 | 0 | 0 | 0 | 1 |
|  | Miyazaki | 6 | 0 | 0 | 0 | 0 | 0 | 1 | 2 | 0 | 0 | 1 | 0 | 0 | 0 | 0 | 12 | 1 | 0 | 0 | 22 | 30 | 0 | 0 | 12 | 0 | 21 | 0 | 0 | 0 | 1 | 0 | 0 | 0 | 0 |
|  | Kagoshima | 2 | 28 | 0 | 0 | 0 | 0 | 0 | 1 | 0 | 0 | 0 | 0 | 0 | 1 | 1 | 7 | 0 | 0 | 0 | 7 | 38 | 0 | 40 | 2 | 4 | 17 | 0 | 0 | 0 | 0 | 0 | 0 | 0 | 0 |
| Okinawa | Okinawa | 0 | 0 | 0 | 0 | 0 | 0 | 0 | 1 | 0 | 0 | 0 | 0 | 0 | 0 | 0 | 1 | 0 | 0 | 0 | 0 | 3 | 0 | 3 | 0 | 0 | 0 | 0 | 0 | 0 | 0 | 0 | 0 | 0 | 0 |

The numbers indicate the number of ticks positive for each *gltA* genotype or the number of ticks analysed in the present study.

Ate, *A. testudinarium*; Dta, *D. taiwanensis*; Hcn, *H. concinna*; Hcg, *H. cornigera*; Hfl, *H. flava*; Hfo, *H. formosensis*; Hja, *H. japonica*; Hhy, *H. hystricis*; Hki, *H. kitaokai*; Hlo, *H. longicornis*, Hme, *H. megaspinosa*; Hye, *H. yeni*; Imo, *I. monospinosus*; Ini, *I. nipponensis*; Iov, *I. ovatus*; Ipe, *I. persulcatus*; Ipa, *I. pavlovskyi*, Ita, *I. tanuki*; Itu, *I. turdus*.

**Supplementary Table S2: Sequence identity with the closest *Rickettsia* species by BLAST analysis.**

| *gltA* genotypes | Tick ID | Tick species | % identity with the closest *Rickettsia* species (Accession number) | | | | | |
| --- | --- | --- | --- | --- | --- | --- | --- | --- |
|  |  |  | *gltA* | *ompA* | *ompB* | *htrA* (17kDa) | *sca4* (geneD) | 16S rRNA |
| G1 | 2021 | *H. megaspinosa* | 99% *R. raoultii* (KY474576) | Not amplified | 97% *R. rhipicephali* (CP013133) | 99% *R. raoultii* (CP019435) | 99% *R. heilongjiangensis* (CP002912) | 99% *R. conorii* (NR074480) |
|  |  |  | 99% *R. raoultii* (MF511249) |  | 97% *R. aeschlimannii* (MF002557) | 99% *R. raoultii* (CP010969) | 99% *R. heilongijiangensis* (AY331396) | 99% *R. conorii* (MF002584) |
|  |  |  | 99% *R. raoultii* (MF511248) |  | 97% *R. amblyommatis* (CP015012) | 99% *R. raoultii* (JX885457) | 99% *R. japonica* (AF155055) | 99% *R. conorii* (KY069267) |
| G2 | 2024 | *H. hystricis* | 99% *R. raoultii* (JQ792120) | Not amplified | 96% *R. aeschlimannii* (MF002557) | 98% *R. raoultii* (CP019435) | 97% *R. heilongijiangensis* (CP002912) | 99% *R. massiliae* (MF002582) |
|  |  |  | 99% *R. raoultii* (JQ792113) |  | 96% *R. amblyommatis* (CP015012) | 98% *R. raoultii* (CP010969) | 97% *R. heilongijiangensis* (AY331396) | 99% *R. massiliae* (KY069266) |
|  |  |  | 99% *R. raoultii* (KY474576) |  | 96% *R. amblyommatis* (KX151487) | 98% *R. raoultii* (JX885457) | 96% *R. slovaca*(MF002531) | 99% *R. massiliae* (CP000683) |
| G3 | 152 | *I. persulcatus* | 100% *C*. R. tarasevichiae (KT899085) | 100% *C.* R. tarasevichiae (KU361217) | Not amplified | 98% *C.* R. tarasevichiae (HF935071) | Not amplified | 100% *C.* R. tarasevichiae (AF503168) |
|  |  |  | 100% *C*. R. tarasevichiae (KT899084) | 100% *C.* R. tarasevichiae (KT119436) |  | 99% *C.* R. tarasevichiae (KP769800) |  | 99% *R. felis* (DQ102712) |
|  |  |  | 100% *C*. R. tarasevichiae (KU310586) | 99% *C.* R. tarasevichiae (KP982901) |  | 99% *C.* R. tarasevichiae (KX365195) |  | 99% *R. rhipicephali* (NR074473) |
| G4 | 434 | *I. persulcatus* | 100% *R. helvetica* (KU310588) | Not amplified | 100% *R. helvetica* (MF163037) | 97% *R. raoultii* (CP019435) | Not amplified | 100% *R. helvetica* (L36212) |
|  |  |  | 100% *R. helvetica* (KT825961) |  | 100% *R. helvetica* (KT835126) | 97% *R. raoultii* (CP010969) |  | 99% *R. felis* (DQ102712) |
|  |  |  | 100% *R. helvetica* (KT825960) |  | 100% *R. helvetica* (KU310591) | 97% *R. raoultii* (JX885457) |  | 99% *R. raoultii* (KY474575) |
| G5 | 61 | *I. persulcatus* | 99% *R. helvetica* (KY488349) | Not amplified | Not amplified | 99% *R. helvetica* (AF181036) | Not amplified | Not amplified |
|  |  |  | 99% *R. helvetica* (KU310588) |  |  | 97% *R. raoultii* (CP019435) |  |  |
|  |  |  | 99% *R. helvetica* (KT825961) |  |  | 97% *R. raoultii* (CP010969) |  |  |
| G5 | 1244 | *I. monospinosus* | 99% *R. helvetica* (KY488349) | Not amplified | 99% *R. helvetica* (MF163037) | 99% *R. helvetica* (AF181036) | 99% *R. helvetica* (AF163009) | 99% *R. helvetica* (L36212) |
|  |  |  | 99% *R. helvetica* (KU310588) |  | 99% *R. helvetica* (KT835126) | 97% *R. raoultii* (CP010969) | 99% *R. asiatica* (DQ110869) | 99% *R. felis* (DQ102712) |
|  |  |  | 99% *R. helvetica* (KT825961) |  | 99% *R. helvetica* (KT835112) | 97% *R. raoultii* (JX885457) | 91% *R. amblyommatis* (CP015012) | 99% *R. raoultii* (KY474575) |
| G6 | 682 | *H. longicornis* | 99% *R. japonica*(KX987343) | 95% *R. heilongjiangensis* (AB473813) | 99% *R. japonica* (AP017588) | 99% *R. rickettsii* (CP018914) | 99% *R. heilongjiangensis* (CP002912) | 99% *R. japonica*(NR074459) |
|  |  |  | 99% *R. japonica*(AP017602) | 95% *R. heilongjiangensis* (AH012829) | 99% *R. japonica* (AP017587) | 99% *R. rickettsii* (CP018913) | 99% *R. heilongjiangensis* (AY331396) | 99% *R. japonica*(KX987315) |
|  |  |  | 99% *R. japonica*(AP017601) | 95% *R. heilongjiangensis* (AF179362) | 99% *R. japonica* (AP017586) | 99% *R. parkeri* (KX018052) | 98% *R. peacockii* (CP001227) | 99% *R. japonica*(KX987314) |
| G7 | 729 | *H. formosensis* | 98% *R. raoultii* (KU723493) | 92% *R. raoultii* (JQ792162) | 97% *R.japonica* (AP017588) | 99% *R. raoultii* (CP019435) | 98% *R. raoultii* (KX506746) | 99% *R. massiliae* (CP003319) |
|  |  |  | 98% *R. raoultii* (KY474576) | 92% *R. raoultii* (JQ792150) | 97% *R.japonica* (AP017587) | 99% *R. raoultii* (CP010969) | 97% *R. raoultii* (KX506747) | 99% *R. massiliae* (MF002582) |
|  |  |  | 98% *R. raoultii* (MF511248) | 92% *R. raoultii* (JQ792137) | 97% *R.japonica* (AP017586) | 99% *R. rickettsii* (JX885457) | 97% *R. raoultii* (JN242188) | 99% *R. massiliae* (KY069266) |
| G8 | 1347 | *A. testudinarium* | 100% *R. tamurae* (AF812551) | 100% *R. tamurae* (DQ103259) | 98% *R. tamurae* (DQ113910) | 100% *R. tamurae* (AB812550) | Not amplified | 100% *R. tamurae* (NR042727) |
|  |  |  | 100% *R. tamurae* (AF394896) | 100% *R. tamurae* (AB114823) | 98% *R. monacensis* (KU961543) | 99% *R. monacensis* (LN794217) |  | 99% *R. monacensis* (LN794217) |
|  |  |  | 99% *R. tamurae* (KT753273) | 99% *R. tamurae* (AB795206) | 98% *R. monacensis* (LN794217) | 99% *R. asembonensis* (KY445736) |  | 99%  *R. monacensis* (NR115686) |
| G9 | 2261 | *H. kitaokai* | 96% *R. raoultii* (JQ792120) | Not amplified | 92% *R. felis* (GU324467) | 96% *R. rhipicephali* (CP003342) | Not amplified | 99% *R. australis* (CP003338) |
|  |  |  | 96% *R. raoultii* (JQ792113) |  | 91% *R. felis* (GU324466) | 96% *R. raoultii* (CP019435) |  | 99% *R. australis* (NR036773) |
|  |  |  | 96% *R. raoultii* (KY474576) |  | 91% *R. felis* (GT835108) | 96% *R. raoultii* (CP019669) |  | 99% *R. australis* (L36101) |
| G10 | 2331 | *I. nipponensis* | 100% *R. monacensis* (KC993860) | 100% *R. monacensis* (JX972178) | 99% *R. monacensis* (KU961543) | 100% *R. monacensis* (LN794217) | Not amplified | 100% *R. monacensis* (KX987306) |
|  |  |  | 100% *R. monacensis* (EU665253) | 100% *R. monacensis* (EU665232) | 99% *R. monacensis* (LN794217) | 99% *R. tamurae* (AB812550) |  | 100% *R. monacensis* (KX987305) |
|  |  |  | 99% *R. monacensis* (KX987342) | 100% *R. monacensis* (KJ588273) | 99% *R. monacensis* (KC137254) | 97% *R. asembonensis* (KY445736) |  | 100% *R. monacensis* (KX987304) |
| G11 | 192 | *H. japonica* | 99% *R. raoultii* (KY474576) | Not amplified | 96% *R. rhipicephali* (CP013133) | 99% *R. raoultii* (CP019435) | 98% *R. heilongijiangensis* (CP002912) | 99% *R. conorii* (NR074480) |
|  |  |  | 99% *R. raoultii* (MF511249) |  | 96% *R. aeschlimannii* (MF002557) | 99% *R. raoultii* (CP010969) | 98% *R. heilongijiangensis* (AY331396) | 99% *R. conorii* (MF002584) |
|  |  |  | 99% *R. raoultii* (MF511248) |  | 96% *R. massiliae*(KT835123) | 99% *R. raoultii* (JX885457) | 98% *R. japonica* (AF155055) | 99% *R. conorii* (KY069267) |
| G11 | 2324 | *H. flava* | 99% *R. raoultii* (KY474576) | Not amplified | Not amplified | 99% *R. raoultii* (CP019435) | Not amplified | Not amplified |
|  |  |  | 99% *R. raoultii* (MF511249) |  |  | 99% *R. raoultii* (CP010969) |  |  |
|  |  |  | 99% *R. raoultii* (MF511248) |  |  | 99% *R. raoultii* (JX885457) |  |  |
| G12 | 2103 | *H. flava* | 99% *R. raoultii* (KY474576) | Not amplified | Not amplified | 99% *R. raoultii* (CP019435) | Not amplified | Not amplified |
|  |  |  | 99% *R. raoultii* (MF511249) |  |  | 99% *R. raoultii* (CP010969) |  |  |
|  |  |  | 99% *R. raoultii* (MF511248) |  |  | 99% *R. raoultii* (JX885457) |  |  |
| G13 | 1277 | *I. ovatus* | 100% *R. asiatica* (AF394901) | Not amplified | 99% *R. helvetica* (KU310591) | 96% *R. raoultii* (CP019435) | Not amplified | 99% *R. asiatica* (NR041840) |
|  |  |  | 99% *R. asiatica* (AB297808) |  | 99% *R. helvetica* (KT825966) | 96% *R. raoultii* (CP010969) |  | 99% *R. helvetica* (L36212) |
|  |  |  | 99% *R. asiatica* (AB297810) |  | 99% *R. helvetica* (KP866151) | 96% *R. raoultii* (JX885457) |  | 99% *R. massiliae* (MF002582) |
| G14 | 1791 | *H. formosensis* | 98% *R. raoultii* (KY474576) | Not amplified | Not amplified | 98% *R. raoultii* (CP019435) | Not amplified | Not amplified |
|  |  |  | 98% *R. raoultii* (MF511249) |  |  | 98% *R. raoultii* (CP010969) |  |  |
|  |  |  | 98% *R. raoultii* (MF511248) |  |  | 98% *R. raoultii* (JX885457) |  |  |
| G15 | 1820 | *H. formosensis* | 98% *R. raoultii* (KY474576) | Not amplified | Not amplified | 97% *R. raoultii* (CP019435) | Not amplified | Not amplified |
|  |  |  | 98% *R. raoultii* (MF511249) |  |  | 97% *R. raoultii* (CP010969) |  |  |
|  |  |  | 98% *R. raoultii* (MF511248) |  |  | 97% *R. raoultii* (JX885457) |  |  |

**Figure S1. The sequence alignment of 15 *gltA* genotypes.** Polymorphic sites are highlighted in yellow.

....|....| ....|....| ....|....| ....|....| ....|....|

5 15 25 35 45

G1(LC379427) ATTGCTAAGA TACCTACCAT CGCCACAATG TCTTATAAAT ATTCTATAGG

G2(LC379428) ATTGCTAAGA TACCTACCAT CGCAGCAATG TCTTATAAAT ATTCTATAGG

G3(LC379429) ATTGCTAAGA TACCGACCAT TGCTGCAATG TCTTATAAAT ATTCTATAGG

G4(LC379430) ATTGCTAAGA TACCTACCAT CGCCGCAATG TCTTATAAAT ATTCTATAGG

G5(LC379431) ATTGCTAAGA TACCTACCAT CGCCGCAATG TCTTATAAAT ATTCTATAGG

G6(LC379433) ATTGCTAAGA TACCTACCAT CGCTGCAATG TCTTATAAAT ATTCTATAGG

G7(LC379434) ATTGCTAAAA TACCTACCAT CGCCGCAATG TCTTATAAAT ATTCTATAGG

G8(LC379435) ATTGCTAAGA TACCTACTAT CGCCGCAATG TCTTATAGAT ATTCTATAGG

G9(LC379436) ATTGCTAAGA TACCTACCAT CGCCGCAATG TCTTATAAAT ATTCTATAGG

G10(LC379437)ATTGCTAAGA TACCTACTAT CGCCGCAATG TCTTATAAAT ATTCTATAGG

G11(LC379438)ATTGCTAAGA TACCTACCAT CGCCACAATG TCTTATAAAT ATTCTATAGG

G12(LC379440)ATTGCTAAGA TACCTACCAT TGCCACAATG TCTTATAAAT ATTCTATAGG

G13(LC379441)ATTGCTAAGA TACCTACCAT CGCCGCAATG TCTTATAAAT ATTCTATAGG

G14(LC379442)ATTGCTAAGA TACATACCAT CGCCGCAATG TCTTATAAAT ATTCTATAGG

G15(LC379443)ATTGCTAAGA TACCTACCAT CGCCGCAATG TCTTATAAAT ATTCCATAGG

....|....| ....|....| ....|....| ....|....| ....|....|

55 65 75 85 95

G1(LC379427) ACAACCGTTT ATTTATCCTG ATAATTCGTT AGATTTTACC GAAAATTTTC

G2(LC379428) ACAACCGTTT ATTTATCCTG ATAATTCGTT AGATTTTACC GAAAATTTTC

G3(LC379429) ACAACCCTTT ATTTACCCTG ATAATTCATT AGATTTTACC GAAAATTTCT

G4(LC379430) ACAACCGTTT ATTTATCCTG ATAATTCGTT AGATTTTACC GAAAATTTTC

G5(LC379431) ACAACCGTTT ATTTATCCTG ATAATTCGTT AGATTTTACC GAAAATTTTC

G6(LC379433) ACAACCGTTT ATTTATCCTG ATAATTCGTT AGATTTTACC GAAAATTTTC

G7(LC379434) ACAACCGTTT ATTTATCCTG ATAATTCGTT AGATTTTACC GAAAATTTTC

G8(LC379435) GCAACCGTTT ATTTATCCTG ATAATTCGTT AGATTTTACC GAAAATTTTC

G9(LC379436) ACAACCGTTT ATTTATCCTG ATAATGCGCT AGATTTTACC GAAAATTTTC

G10(LC379437)GCAACCGTTT ATTTATCCTG ATAATTCGTT AGATTTTACC GAAAATTTTC

G11(LC379438)ACAACCGTTT ATTTATCCTG ATAATTCGTT AGATTTTACC GAAAATTTTC

G12(LC379440)ACAACCGTTT ATTTATCCTG ATAATTCGTT AGATTTTACC GAAAATTTTC

G13(LC379441)ACAACCGTTT ATTTATCCTG ATAATTCGTT AGATTTTACC GAAAATTTTC

G14(LC379442)ACAACCGTTT ATTTATCCTG ATAATTCGTT AGATTTTACT GAAAATTTTC

G15(LC379443)ACAACCGTTT ATTTATCCTG ATAATTCGTT AGATTTTACT GAAAATTTTC

....|....| ....|....| ....|....| ....|....| ....|....|

105 115 125 135 145

G1(LC379427) TGCATATGAT GTTTGCAATG CCTTGTACGA AATATAAAGT AAATCCAATA

G2(LC379428) TGCATATGAT GTTTGCAACG CCTTGTACGA AATATAAAGT AAATCCAATA

G3(LC379429) TACGCATGAT GTTTGCAACA CCTTGTACGA AATATGAAGT AAATCCGGTA

G4(LC379430) TGCATATGAT GTTTGCAACG CCTTGTACGA AATATAAAGT AAATCCAATA

G5(LC379431) TGCATATGAT GTTTGCAACG CCTTGTACGA AATATAAAGT AAATCCAATA

G6(LC379433) TGCATATGAT GTTTGCAACG CCTTGTACGA AATATACAGT AAATCCAATA

G7(LC379434) TGCATATGAT GTTTGCAACG CCTTGTACGA AATATAAAGT AAATCCAATA

G8(LC379435) TGCATATGAT GTTTGCAACG CATTGTACAA AATATAAAGT AAATCCAATA

G9(LC379436) TGCATATGAT GTTTGCAACG CCTTGTACGC AATATAAAGT AAATCCAATA

G10(LC379437)TGCATATGAT GTTTACAACG CATTGTACGA AATATAAAGT AAATCCAATA

G11(LC379438)TGCATATGAT GTTTGCAATG CCTTGTACGA AATATAAAGT AAATCCAATA

G12(LC379440)TGCATATGAT GTTTGCAATG CCTTGTACGA AATATAAAGT AAATCCAATA

G13(LC379441)TGCATATGAT GTTTGCAATG CCTTGTACGA AATATAAAGT AAATCCAATA

G14(LC379442)TGCATATGAT GTTTGCAACG CCTTGTACGA AATATAAAGT AAATCCAATA

G15(LC379443)TGCATATGAT GTTTGCAACG CCTGGTACGA AATATAAAGT AAATCCAATA

....|....| ....|....| ....|....| ....|....| ....|....|

155 165 175 185 195

G1(LC379427) ATAAAAAATG CTCTTAATAA GATATTTATC CTACATGCCG ATCATGAGCA

G2(LC379428) ATAAAAAAGG CTCTTAATAA GATATTTATC CTACATGCCG ATCACGAGCA

G3(LC379429) ATAAAAAATG CTCTTAATAA GATATTTATT CTGCATGCCG ACCACGAGCA

G4(LC379430) ATAAAAAATG CTCTTAATAA GATATTTATC CTACATGCCG ATCATGAGCA

G5(LC379431) ATAAAAAATG CTCTTAATAA GATATTTATC CTACATGCCG ATCATGAGCA

G6(LC379433) ATAAAAAATG CTCTTAATAA GATATTTATC CTACATGCCG ATCATGAGCA

G7(LC379434) ATAAAAAATG CTCTTAATAA GATATTTATC CTACATGCCG ATCATGAGCA

G8(LC379435) ATAAAAAATG CTCTTAATAA GATATTTATC CTACATGCAG ACCATGAGCA

G9(LC379436) ATAACAAATG CTCTTAATAA GATATTTATC CTACATGCTG ATCATGAGCA

G10(LC379437)ATAAAAAATG CTCTTAATAA GATATTTATC CTACATGCAG ACCATGAGCA

G11(LC379438)ATAAAAAATG CTCTTAATAA GATATTTATC CTACATGCCG ATCATGAGCA

G12(LC379440)ATAAAAAATG CTCTTAATAA GATATTTATC CTACATGCCG ATCATGAGCA

G13(LC379441)ATAAAAAATG CTCTTAATAA GATATTTATC CTACATGCCG ATCATGAGCA

G14(LC379442)ATAAAAAATG CTCTTAATAA GATATTTATC CTACATGCCG ATCATGAGCA

G15(LC379443)ATAAAAAATG CTCTTAATAA GATATTTATC CTACATGCCG ATCATGAGCA

....|....| ....|....| ....|....| ....|....| ....|....|

205 215 225 235 245

G1(LC379427) GAATGCTTCT ACTTCAACAG TCCGAATTGC CGGCTCATCC GGAGCTAACC

G2(LC379428) GAATGCTTCT ACTTCAACAG TCCGAATTGC CAGCTCATCC GGAGCTAATC

G3(LC379429) GAATGCTTCT ACCTCAACAG TAAGAATTGC CGGTTCATCC GGAGCTAATC

G4(LC379430) GAATGCTTCT ACTTCAACAG TCCGAATTGC CGGCTCATCT GGAGCTAACC

G5(LC379431) GAATGCTTCT ACTTCAACAG TCCGCATTGC CGGCTCATCT GGAGCTAACC

G6(LC379433) GAATGCTTCT ACTTCAACAG TCCGAATTGC CGGCTCATCC GGAGCTAACC

G7(LC379434) GAATGCTTCT ACTTCAACAG TCCGAATTGC CGGCTCATCT GGAGCTAACC

G8(LC379435) GAATGCTTCT ACCTCAACAG TTCGAATTGC CGGCTCATCC GGAGCTAACC

G9(LC379436) GAATGCTTCT ACTTCAACTG TCCGAATTGC CGGCTCATCT GGAGCTAATC

G10(LC379437)GAATGCTTCT ACCTCAACAG TTCGAATTGC CGGCTCATCC GGAGCTAACC

G11(LC379438)GAATGCTTCT ACTTCAACAG TCCGAATTGC CGGCTCATCC GGAGCTAACC

G12(LC379440)GAATGCTTCT ACTTCAACAG TCCGAATTGC CGGCTCATCC GGAGCTAACC

G13(LC379441)GAATGCTTCT ACTTCAACAG TCCGAATTGC CGGCTCATCC GGAGCTAACC

G14(LC379442)GAATGCTTCT ACTTCAACAG TCCGAATTGC CGGCTCATCC GGAGCTAACC

G15(LC379443)GAATGCTTCC ACTTCAACAG TCCGAATTGC CGGCTCATCC GGAGCTAACC

....|....| ....|....| ....|....| ....|....| ....|....|

255 265 275 285 295

G1(LC379427) CTTTTGCTTG TATTAGCACG GGTATTGCCT CACTTTGGGG ACCTGCTCAC

G2(LC379428) CTTTTGCTTG TATTAGCACG GGTATTGCCT CACTTTGGGG ACCTGCTCAC

G3(LC379429) CTTTTGCTTG TATTAGTACA GGTATTGCCT CACTTTGGGG GCCTGCTCAC

G4(LC379430) CTTTTGCTTG TATTAGTACG GGTATTGCAT CACTTTGGGG ACCTGCTCAC

G5(LC379431) CTTTTGCTTG TATTAGTACG GGTATTGCAT CACTTTGGGG ACCTGCTCAC

G6(LC379433) CTTTTGCTTG TATTAGCACG GGTATTGCCT CACTTTGGGG ACCTGCTCAC

G7(LC379434) CTTTTGCTTG TATTAGCACG GGTATTGCCG CACTTTGGGG ACCTGCTCAC

G8(LC379435) CTTTTGCTTG TATTAGCACG GGTATTGCCT CACTTTGGGG ACCTGCTCAC

G9(LC379436) CTTTTGCTTG TATTAGCACG GGCATTGCTT CACTTTGGGG ACCTGCTCAC

G10(LC379437)CTTTTGCTTG TATTAGCACG GGTATTGCCT CACTTTGGGG ACCTGCTCAC

G11(LC379438)CTTTTGCTTG TATTAGCACG GGTATTGCCT CACTTTGGGG ACCTGCTCAC

G12(LC379440)CTTTTGCTTG TATTAGCACG GGTATTGCCT CACTTTGGGG ACCTGCTCAC

G13(LC379441)CTTTTGCTTG TATTAGTACG GGTATTGCAT CACTTTGGGG ACCTGCTCAC

G14(LC379442)CTTTTGCTTG TATTAGCACG GGTATTGCCT CACTTTGGGG ACCTGCTCAC

G15(LC379443)CTTTTGCTTG TATTAGCACG GGTATTGCCT CACTTTGGGG ACCTGCTCAC

....|....| ....|....| ....|....| ....|....| ....|....|

305 315 325 335 345

G1(LC379427) GGCGGGGCTA ATGAAGCGGT AATAAATATG CTTAAAGAAA TCGGTAGTTC

G2(LC379428) GGCGGGGCTA ATGAAGCGGT AATAAATATG CTTAAAGAAA TCGGTAGTTC

G3(LC379429) GGCGGGGCTA ATGAAGCGGT AATAAATATG CTTAAAGAAA TCGGTAGTTC

G4(LC379430) GGTGGGGCTA ATGAAGCGGT AATAAATATG CTTAAAGAAA TTGGTAGTTC

G5(LC379431) GGCGGGGCTA ATGAAGCGGT AATAAATATG CTTAAAGAAA TTGGTAGTTC

G6(LC379433) GGTGGGGCTA ATGAAGCGGT AATAAATATG CTTAAAGAAA TCGGTAGTTC

G7(LC379434) GGCGGGGCTA ATGAAGCGGT AATAAATATG CTTAAAGAAA TCGGTAGTTC

G8(LC379435) GGCGGGGCTA ATGAAGCGGT AATAAATATG CTTAAAGAAA TCGGTAGTTC

G9(LC379436) GGTGGGGCTA ATGAAGCGGT AACAAATATG CTTAAAGAAA TCGGTAGTTC

G10(LC379437)GGCGGGGCTA ATGAAGCGGT AATAAATATG CTTAAAGAAA TCGGTAGTTC

G11(LC379438)GGCGGGGCTA ATGAAGCGGT AATAAATATG CTTAAAGAAA TCGGTAGTTC

G12(LC379440)GGCGGGGCTA ATGAAGCGGT AATAAATATG CTTAAAGAAA TCGGTAGTTC

G13(LC379441)GGCGGGGCTA ATGAAGCGGT AATAAATATG CTTAAAGAAA TCGGTAGTTC

G14(LC379442)GGCGGGGCTA ATGGAGCGGA AATAAATATG CTTAAAGAAA TCAGTAGTTC

G15(LC379443)GGCGGGGCTA ATGGAGCGGT AATAAATATG CTTAAAGAAA TCAGTAGTTC

....|....| ....|....| ....|....| ....|....| ....|....|

355 365 375 385 395

G1(LC379427) TGAGTATATT ACTAAATATA TAGCTAAAGC TAAGGATAAA AATGATCCAT

G2(LC379428) TGAGTATATT CCTAAATATA TAGCTAAAGC TAAGGATAAA AATGATCCAT

G3(LC379429) TGAGAATATC CCTAAATATA TAGCTAAAGC TAAAGATAAA GATGACCCGT

G4(LC379430) TGAGAATATC CCTAAATATA TAGCTAAAGC TAAGGATAAA AATGATCCGT

G5(LC379431) TGAGAATATC CCTAAATATA TAGCTAAAGC TAAGGATAAA AATGATCCGT

G6(LC379433) TGAGTATATT CCTCGATATA TAGCTAAAGC TAAGGATAAA AATGATCCAT

G7(LC379434) GGAGTATATT CCTAAATATA TAGCTAAAGC TAAGGATAAA AATGATCCAT

G8(LC379435) TGAGAATATC CCTAAATATA TAGCTAAAGC TAAGGATAAA AATGATCCGT

G9(LC379436) TGAGAATATC CCTAAATATA TAGCTAAAGC TAAGGATAAA AATGATCCGT

G10(LC379437)TGAGAATATC CCTAAATATA TAGCTAAAGC TAAGGATAAA AATGATCCGT

G11(LC379438)TGAGTATATT CCTAAATATA TAGCTAAAGC TAAGGATAAA AATGATCCAT

G12(LC379440)TGAGTATATT CCTAAATATA TAGCTAAAGC TAAGGATAAA AATGATCCAT

G13(LC379441)TGAGAATATC CCTAAATATA TAGCTAAAGC TAAGGATAAA AATGATCCAT

G14(LC379442)TGAGTATATT CCTAAATATA TAGTTAAAGC TACGGATAAA AATGATCCAT

G15(LC379443)TGAGTATATT CCTAAATATA TAGTTAAAGC TAAGGATAAA AATGATCCAT

....|....| ....|....| ....|....| ....|....| ....|....|

405 415 425 435 445

G1(LC379427) TTAGGTTAAT GGGTTTTGGT CATCGTGTAT ATAAAAACTA TGACCCGCGT

G2(LC379428) TTAGGTTAAT GGGTTTTGGT CATCGTGTAT ATAAAAACTA TGACCCGCGT

G3(LC379429) TTAGGTTAAT GGGCTTCGGT CATCGTGTCT ATAAAAACTA TGACCCACGT

G4(LC379430) TTAGGTTAAT AGGCTTCGGT CATCGTGTAT ATAAAAACTA CGATCCACGT

G5(LC379431) TTAGGTTAAT AGGCTTCGGT CATCGTGTAT ATAAAAACTA CGATCCACGT

G6(LC379433) TTAGGTTAAT GGGTTTTGGT CATCGTGTAT ATAAAAACTA TGACCCGCGT

G7(LC379434) TTAGGTTAAT GGGTTTTGGT CATCGTGTGT ATAAAAACTA TGATCCGCGT

G8(LC379435) TTAGGTTAAT GGGTTTCGGT CATCGTGTAT ATAAAAACTA TGACCCGCGT

G9(LC379436) TTAGGTTAAT GGGTTTTGGT CATCGTGTCT ATAAAAACTA TGACCCGCGT

G10(LC379437)TTAGGTTAAT GGGTTTCGGT CATCGTGTAT ATAAAAACTA TGACCCGCGT

G11(LC379438)TTAGGTTAAT GGGTTTTGGT CATCGTGTAT ATAAAAACTA TGACCCGCGT

G12(LC379440)TTAGGTTAAT GGGTTTTGGT CATCGTGTAT ATAAAAACTA TGACCCGCGT

G13(LC379441)TTAGGTTAAT AGGCTTCGGT CATCGTGTAT ATAAAAACTA CGATCCACGT

G14(LC379442)TTAGGTTAAT CGGTTTTGGT CATCGTGTAT ATAAAAACTA TGACCCGCGT

G15(LC379443)TTAGGTTAAT GGGTTTTGGT CATCGTGTAT ATAAAAACTA TGACCCGCGT

....|....| ....|....| ....|....| ....|....| ....|....|

455 465 475 485 495

G1(LC379427) GCCGCAGTAC TGAAAGAAAC GTGCAAAGAA GTATTAAAGG AACTCGGGCA

G2(LC379428) GCCGCAGTAC TTAAAGAAAC GTGCAAAGAA GTATTAAAGG AACTCAGGCA

G3(LC379429) GCCGCAGTAC TTAAAGAAAC TTGTAAGGAA GTATTAAAGG AACTTGGACA

G4(LC379430) GCCGCAGTAC TTAAAGAAAC TTGTAAGGAA GTATTAAAGG AACTCGGACA

G5(LC379431) GCCGCAGTAC TTAAAGAAAC TTGTAAGGAA GTATTAAAGG AACTCGGACA

G6(LC379433) GCCGCAGTAC TTAAAGAAAC GTGCAAAGAA GTATTAAAGG AACTCGGGCA

G7(LC379434) GCTGCAGTAC TTAAAGAAAC GTGCAAAGAA GTATTAAAGG AACTCGGGCA

G8(LC379435) GCCGCAGTAC TTAAAGAAAC GTGCAAAGAA GTGTTAAAGG AACTCGGACA

G9(LC379436) GCTGCAGTAC TTAAAGAAAC GTGTAAAGAA GTACTAAAGG AACTCGGGCA

G10(LC379437)GCCGCAGTAC TTAAAGAAAC GTGCAAAGAA GTATTAAAGG AACTCGAACA

G11(LC379438)GCCGCAGTAC TGAAAGAAAC GTGCAAAGAA GTATTAAAGG AACTCGGGCA

G12(LC379440)GCCGCAGTAC TGAAAGAAAC GTGCAAAGAA GTATTAAAGG AACTCGGGCA

G13(LC379441)GCCGCAGTAC TTAAAGAAAC TTGTAAGGAA GTATTAAAGG AACTCGGACA

G14(LC379442)GCCGCAGTAC TTAAAGAAAC GTGCAAAGAA GTATTAAAGG AACTTGGACA

G15(LC379443)GCCGCAGTAC TTAAAGAAAC GTGCAAAGAA GTATTAAAGG AACTTCGGCA

....|....| ....|....| ....|....| ....|..

505 515 525 535

G1(LC379427) GCTAGACAAC AATCCGCTCT TACAAATAGC AATAGAA

G2(LC379428) GCTAGACAAC AATCCGCTCT TACAAATAGC AATAGAA

G3(LC379429) GCTAGAAAAC AATCCGCTCT TACAAATAGC AATAGAA

G4(LC379430) GCTAGAAAAC AATCCGCTCT TACAAATAGC AATAGAA

G5(LC379431) GCTAGAAAAC AATCCGCTCT TACAAATAGC AATAGAA

G6(LC379433) GCTAGACAAC AATCCGCTCT TACAAATAGC AATAGAA

G7(LC379434) GCTAGACAAT AATCCACTCT TACAAATAGC AATAGAA

G8(LC379435) GTTAGAAAAT AATCCACTTT TACAAATAGC AATAGAA

G9(LC379436) GCTAGAAAAC AATCCGCTCT TACAAATAGC AATAGAA

G10(LC379437)GTTAGAAAAT AATCCACTTT TACAAATAGC AATAGAA

G11(LC379438)GCTAGACAAC AATCCGCTCT TACAAATAGC AATAGAA

G12(LC379440)GCTAGACAAC AATCCGCTCT TACAAATAGC AATAGAA

G13(LC379441)GCTAGAAAAC AATCCGCTCT TACAAATAGC AATAGAA

G14(LC379442)GCTAGACAAC AATCCGCTCT TACAAATAGC AATAGAA

G15(LC379443)GCTAGACAAC AATCCGCTCT TACAAATAGC AATAGAA
